# Novel Efficient Lipid-Based Delivery Systems Enable a Delayed Uptake and Sustained Expression of mRNA in Human Cells and Mouse Tissues

**DOI:** 10.3390/pharmaceutics16050684

**Published:** 2024-05-19

**Authors:** Artem G. Fedorovskiy, Denis N. Antropov, Anton S. Dome, Pavel A. Puchkov, Daria M. Makarova, Maria V. Konopleva, Anastasiya M. Matveeva, Eugenia A. Panova, Elena V. Shmendel, Mikhail A. Maslov, Sergey E. Dmitriev, Grigory A. Stepanov, Oleg V. Markov

**Affiliations:** 1 Belozersky Institute of Physico-Chemical Biology, Department of Materials Science, Faculty of Bioengineering and Bioinformatics, Lomonosov Moscow State University, 119234 Moscow, Russia; fed@belozersky.msu.ru (A.G.F.); maria-konopleva@rambler.ru (M.V.K.); panova.evgenia@gmail.com (E.A.P.); 2 Lomonosov Institute of Fine Chemical Technologies, MIREA-Russian Technological University, 119571 Moscow, Russia; puchkov_p@mirea.ru (P.A.P.); das.kozl@yandex.ru (D.M.M.); shmendel@mirea.ru (E.V.S.); maslov_m@mirea.ru (M.A.M.); 3 Institute of Chemical Biology and Fundamental Medicine, Siberian Branch of the Russian Academy of Sciences, 630090 Novosibirsk, Russia; antr0povdn@yandex.ru (D.N.A.); domeanton@yandex.ru (A.S.D.); anastasiya.maatveeva@gmail.com (A.M.M.); stepanovga@niboch.nsc.ru (G.A.S.); 4 Federal State Budget Institution “National Research Centre for Epidemiology and Microbiology Named after Honorary Academician N.F. Gamaleya” of the Ministry of Health of the Russian Federation, 123098 Moscow, Russia; 5 Moscow Institute of Physics and Technology, 141701 Dolgoprudny, Russia

**Keywords:** mRNA transfection, lipofection, lipid nanoparticles, LNP, cationic lipid, ionizable lipid, liposomes, lipoplexes, mRNA vaccines, mRNA therapy

## Abstract

Over the past decade, mRNA-based therapy has displayed significant promise in a wide range of clinical applications. The most striking example of the leap in the development of mRNA technologies was the mass vaccination against COVID-19 during the pandemic. The emergence of large-scale technology and positive experience of mRNA immunization sparked the development of antiviral and anti-cancer mRNA vaccines as well as therapeutic mRNA agents for genetic and other diseases. To facilitate mRNA delivery, lipid nanoparticles (LNPs) have been successfully employed. However, the diverse use of mRNA therapeutic approaches requires the development of adaptable LNP delivery systems that can control the kinetics of mRNA uptake and expression in target cells. Here, we report effective mRNA delivery into cultured mammalian cells (HEK293T, HeLa, DC2.4) and living mouse muscle tissues by liposomes containing either 1,26-bis(cholest-5-en-3β-yloxycarbonylamino)-7,11,16,20-tetraazahexacosane tetrahydrochloride (2X3) or the newly applied 1,30-bis(cholest-5-en-3β-yloxycarbonylamino)-9,13,18,22-tetraaza-3,6,25,28-tetraoxatriacontane tetrahydrochloride (2X7) cationic lipids. Using end-point and real-time monitoring of Fluc mRNA expression, we showed that these LNPs exhibited an unusually delayed (of over 10 h in the case of the 2X7-based system) but had highly efficient and prolonged reporter activity in cells. Accordingly, both LNP formulations decorated with 1,2-distearoyl-*sn*-glycero-3-phosphoethanolamine-*N*-[amino(polyethylene glycol)-2000] (DSPE-PEG_2000_) provided efficient luciferase production in mice, peaking on day 3 after intramuscular injection. Notably, the bioluminescence was observed only at the site of injection in caudal thigh muscles, thereby demonstrating local expression of the model gene of interest. The developed mRNA delivery systems hold promise for prophylactic applications, where sustained synthesis of defensive proteins is required, and open doors to new possibilities in mRNA-based therapies.

## 1. Introduction

In recent years, mRNA therapy has shown considerable potential in the treatment of various diseases [1,2,3,4,5,6]. The rapid development and effective use of mRNA-based vaccines in combating the COVID-19 pandemic [7,8,9,10] has further propelled the adoption of this technology in various clinical directions, including the prevention of infectious and treatment of hereditary diseases, monoclonal antibody production, and cancer immunotherapy [2,11,12,13,14].

The most clinically advanced vehicles to deliver mRNA into mammalian cells are lipid nanoparticles (LNPs) composed of cationic or ionizable lipids combined with neutral helper lipids and other auxiliary components [14,15,16]. LNPs are adsorbed to the cell surface and uptaken by the cell, followed by mRNA release into the cytoplasm [17]. However, the molecular mechanisms underlying these processes are not fully understood.

Liposomes and liposome-mRNA complexes (lipoplexes) can likely be internalized in multiple ways, including clathrin or caveolin-mediated endocytosis, macropinocytosis, and phagocytosis, as well as by direct fusion with the cell membrane [15,18,19,20,21]. Typically, the uptaken RNA-LNPs are entrapped in early endosomes and, after they mature to late endosomes and then to endolysosomes, degraded by acidic pH and endolysosomal enzymes [22,23,24,25,26]. Additionally, they can be returned back to the extracellular space via exocytic (recycling) route [26,27,28,29]. Both of these pathways mark non-productive trafficking. Finally, the cargo can escape the endosomal compartment at a poorly determined stage through osmotic swelling, membrane fusion, or membrane destabilization [19]. This brings mRNA to the cytoplasmic translation machinery, thus marking functional delivery of the cargo [17,30]. Yet, endosomal escape occurs with only a small fraction of RNA molecules, representing the bottleneck for efficient transfection [18,22,23,24,25,26,31]. 

The efficiency of mRNA functional delivery is largely determined by the chemical structures of the lipids used to form LNPs, which usually consists of a mixture of ionizable/cationic and helper lipids, sometimes with cholesterol addition, and usually supplemented with a PEG lipid for in vivo delivery [15,32,33,34]. A fair number of cationic and ionizable lipids have been synthesized and utilized as effective RNA carriers [14,15,16,31,33,35,36,37,38,39]. LNP attributes such as size, charge, internal structure, stability, and affinity to molecules exposed on the cell membrane also heavily influence RNA delivery and intracellular protein production in cultured cells and animal tissues (see [33,40,41,42] and references therein).

While numerous studies have focused on the efficiency of mRNA-LNP uptake and protein production, the kinetics underlying these processes remain poorly understood. Some observations (primarily made on short RNAs of less than 24 nt) suggest that a substantial proportion of LNPs are internalized by cells within one hour following transfection [24,43,44,45,46,47,48] or, in some instances, within a few hours [43,49,50]. Furthermore, RNA release from endosomes is reported to occur approximately 5–15 min following endocytosis [23,51]. However, there is a paucity of research investigating the expression kinetics of delivered mRNAs at high minute-scaled resolution over both short and long time intervals.

**Figure 1:**
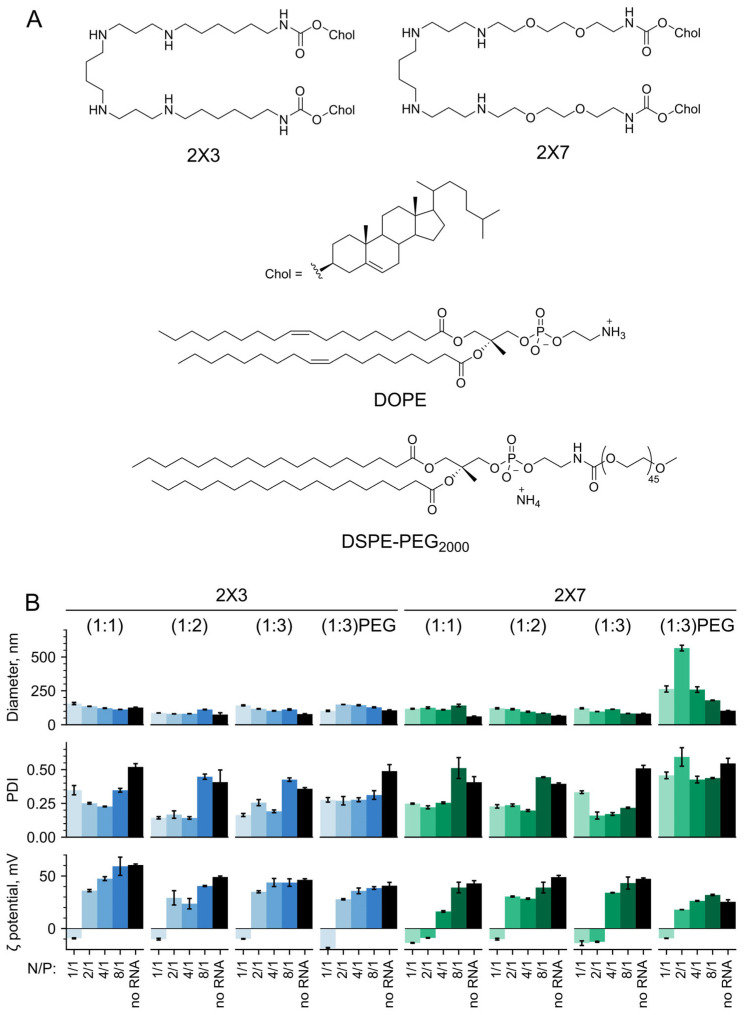
Structures of the lipidic components of cationic liposomes used for mRNA delivery in this study and physicochemical characteristics of the 2X3- (blue bars) and 2X7- (green bars) containing liposomes and lipoplexes. (**A**) Cationic liposomes composed of cationic lipid 2X3 or 2X7 and helper lipid DOPE at different molar ratios. For the in vivo experiment, 2X3-DOPE and 2X7-DOPE liposomes were decorated with DSPE-PEG_2000_ (2% mol). (**B**) Hydrodynamic diameters, PDIs, and ζ-potentials of liposomes and their lipoplexes with mRNA. All experimental points were run in triplicates. Data are represented as mean ± SD. PDI, polydispersity index.

The diverse applications of therapeutic nucleic acids in biology and medicine require the development and evaluation of novel effective delivery systems with a broad range of biological properties. Previously, we developed two spermine- and cholesterol-containing cationic gemini lipids (Figure 1A): 1,26-bis(cholest-5-en-3beta- yloxycarbonylamino)-7,11,16,20-tetraazahexacosane tetrahydrochloride (2X3), which provided efficient delivery of nucleic acids into animal cells and tissues [52,53,54], and 1,30-bis(cholest-5-en-3β-yloxycarbonylamino)-9,13,18,22-tetraaza-3,6,25,28-tetraoxatriacontane tetrahydrochloride (2X7), which is more soluble in biological fluids [55]. The 2X7 lipid differs from the parent 2X3 lipid by length and hydrophilicity of spacer groups linking hydrophobic cholesterol and positively charged spermine domains.

Here, we employ continuous monitoring of reporter expression in living mammalian cells in vitro and in living mouse tissues in vivo to comprehensively characterize the kinetics of functional mRNA delivery by liposomes containing either 2X3 or 2X7 cationic lipid, combined with a helper lipid, 1,2-dioleoyl-*sn*-glycero-3-phosphoethanolamine (DOPE) (in a range from 50 to 75%). The use of DOPE, a helper lipid, in the formulation of cationic liposomes [56,57,58] enhances gene delivery efficiency by facilitating the timely release of nucleic acids through the destabilization of endosomal membranes at a decrease in pH [34]. Notably, our study reveals an exceptional and unprecedented delay (>4 h and >10 h in the case of 2X3 and 2X7, respectively) in product accumulation, followed by highly efficient and prolonged reporter activity, which strikingly contrasts with the mRNA expression profile observed with other delivery systems [59,60,61]. Furthermore, both 2X3 and 2X7-based lipoplexes containing DSPE-PEG_2000_-component demonstrate sustained and efficient luciferase production in mice for up to 9 days, with enzyme activity exhibiting a progressive increase up to day 3 following intramuscular injection. We presented a detailed description of the physicochemical properties of lipoplexes, which helped explain the properties of the resulting delivery systems. The peculiar properties of the 2X7-based LNPs can be used for therapeutic interventions requiring prolonged and temporally controlled mRNA expression.

## 2. Materials and Methods

### 2.1. Preparation of Cationic Liposomes

All cationic liposomes were prepared via the hydrating thin lipid film method [62]. Briefly, a solution of 1,26-bis(cholest-5-en-3β-yloxycarbonylamino)-7,11,16,20-tetraazahexacosane tetrahydrochloride (2X3) [63] or 1,30-bis(cholest-5-en-3β-yloxy-carbonylamino)-9,13,18,22-tetraaza-3,6,25,28-tetraoxatriacontane tetrahydrochloride (2X7) [55] in a mixture of CHCl_3_–CH_3_OH (5:1 vol.) was added to a solution of 1,2-dioleoyl-*sn*-glycero-3-phosphoethanolamine (DOPE, Avanti Polar Lipids, Alabaster, AL, USA) in CHCl_3_ at molar ratios of 1:1, 1:2, and 1:3 and gently stirred. Notably, the predicted pK*_as_* of both 2X3 and 2X7 lipids are about 7.8. To obtain PEG-containing cationic liposomes, a solution of DSPE-PEG_2000_ (Lipoid, Ludwigshafen, Germany) (2% mol.) in CHCl_3_ was added to the solution 2X3-DOPE and 2X7-DOPE at molar ratios of 1:2 and 1:3. Organic solvents were removed in vacuo, and the lipid film obtained was dried for 4 h at 0.1 Torr to remove the residual organic solvent. Then, it was hydrated using deionized sterile water (Milli-Q, Burlington, MA, USA) at 4 °C overnight. The liposomal dispersion was sonicated for 15 min at 75 °C in a bath-type sonicator (Bandelin Sonorex Digitec DT 52H, Berlin, Germany), filtered (0.45 μm Chromafil^®^ CA-45/25; Macherey–Nagel, Düren, Germany), flushed with argon, and stored at 4 °C. In the resulting dispersion, the cationic lipid concentration was 1 mM.

### 2.2. In Vitro Transcription

m^7^G-capped reporter mRNAs were prepared by in vitro transcription using the “Anti-Reverse Cap Analog (ARCA)-mRNA-20” kit (Biolabmix, Novosibirsk, Russia). In the transcription mixture, uridine-5’-triphosphate (UTP) was completely replaced by N1-methylpseudouridine-5’-triphosphate (m^1^ΨTP) (Biolabmix, Novosibirsk, Russia) to reduce transcript immunogenicity. Following RNA synthesis, the DNA template was removed with DNase I (Thermo Fisher Scientific, Waltham, MA, USA). The transcripts obtained were treated by Arctic thermolabile alkaline phosphatase (Biolabmix, Novosibirsk, Russia) to remove 5′-triphosphates from uncapped mRNA products. For experiments involving physicochemical characterization of the lipoplexes, end-point in vitro transfection, and in vivo delivery, the mRNAs encoding mKate2 and firefly luciferase (Fluc) contained the untranslated regions (UTRs), 5′-UTR-4 [64] and 3′-UTR AES-mtRNR1 [65], and were synthesized using the corresponding linearized plasmids (pCMV6_synth_AGG_mKate2 and pCMV6_synth_AGG_luc2, respectively) as templates, followed by 3′-polyadenylation of the transcripts by Poly(A) polymerase (New England Biolabs, Ipswich, MA, USA). To produce enhanced green fluorescent protein (EGFP) encoding mRNA, a PCR product was obtained from pEGFP-C1 plasmid (Clontech, San Jose, CA, USA) using the primers CGCTGTAATACGACTCACTATAGGGAAATAAGAGA GAAAAGAAGAGTAAGAAGAAATATAAGAGCCAAGATGGTAGCAAGGGCGAGGAG and (T)_50_GTGATGCTATTGCTTTATTTGTAACC. For continuous (“real-time”) luciferase measurements, the constructed Fluc containing the human HBA1 5′ and 3′ UTRs, the codon-optimized Fluc coding region (Fluc2), and the plasmid-encoded poly(A) tail was used, as described previously [61]. The RNA products were purified either via a silica spin column-based method using the DR kit (Biolabmix, Novosibirsk, Russia) or by LiCl precipitation, dissolved in deionized sterile diethyl pyrocarbonate (DEPC)-treated water (Biolabmix, Novosibirsk, Russia), and checked for integrity via denaturing polyacrylamide gel electrophoresis [61,66].

### 2.3. Size and Zeta Potential Measurement

Lipoplexes were preliminarily formed by mixing equal (25 μL) volumes of the RNA and the liposomes taken at appropriate concentrations in Milli-Q water, PBS buffer or saline (154 mM sodium chloride). The lipoplex formation was carried out for 20 min at 25 °C. Subsequently, the lipoplexes were diluted in 1 mL of DEPC-treated water. To measure the physicochemical parameters, 1 mL of lipoplex or liposome suspension was placed into the folded capillary cuvette DTS1070 (Malvern Instruments, Malvern, UK). The size and polydispersity index (PDI) of lipoplexes were measured in 3 biological replicates by dynamic light scattering (DLS) using the Malvern Zetasizer Nano (Malvern Instruments, Malvern, UK) at a 173° scattering angle and a temperature of 25 °C. The measurements were performed using Malvern’s Zetasizer v7.11 software (Malvern Instruments, Malvern, UK). A viscosity of 0.8872 centipoises (cP) and refractive index (RI) of 1.330 for the dispersant and an RI of 1.020 and absorption of 1.335 for the material in suspension were set on the software. An equilibration time of 30 s was set before the total measurement. Zeta potential was measured at 25 °C in 3 biological replicates. Before measurement, the equilibration time of 120 s was set. Each measurement was paused for 30 s before the next one.

AFM images were captured in ambient air. Sample preparation for AFM was as follows: (1) dilution of the samples to desired concentration; (2) deposition of 6 μL of a sample onto a freshly prepared mica slide (1 × 1 cm) for adsorption for 60 s; (3) rinsing with 100–1000 μL of Milli-Q water; and (4) drying the specimen with a gentle argon stream. Images were acquired on a Multimode 8 (Bruker, Bremen, Germany) atomic force microscope in “ScanAsyst in Air” mode using ScanAsyst-Air probes (Bruker, Germany) or in tapping mode with a diamondlike carbon NSG-10 series AFM cantilever (NT-MDT, Zelenograd, Russia) with a tip curvature radius of 1–3 nm. Images were processed, prepared and analyzed using Gwyddion software v.2.6.0.

### 2.4. Gel Retardation Assay

To evaluate the packaging efficiency of lipoplexes, a gel retardation assay was conducted. In this assay, 0.5 µg of mKate2 mRNA diluted in PBS (15 μL) were mixed with PBS-diluted 2X3-DOPE or 2X7-DOPE liposomes taken at appropriate concentrations corresponding to particular lipid nitrogen to RNA phosphate (N/P) ratios (15 μL). Lipoplexes were formed for 20 min at 25 °C. To assess lipoplex formation, the samples were combined with 4× loading dye (Biolabmix, Novosibirsk, Russia) and loaded on a 1.5% agarose gel. After running for 20 min at 120V, 200 mA, the gel was visualized using Molecular Imager GelDoc XR+ (Bio-Rad Laboratories Inc., Hercules, CA, USA). The mobility of mRNA was visualized by ethidium bromide staining.

### 2.5. Cell Culture

The human HEK293T (CRL-3216), HEK293T/17 (CRL-11268), HeLa (CCL-2) obtained from ATCC and mouse DC2.4 (SCC142, Millipore, Burlington, MA, USA) cell lines maintained at 37 °C in a humidified atmosphere with 5% CO_2_. HEK293T and HeLa cells were cultured in DMEM (Gibco, Waltham, MA, USA) containing alanyl-glutamine (Paneco, Moscow, Russia) and supplemented with 10% FBS (HyClone, Logan, UT, USA) along with penicillin and streptomycin (Paneco, Moscow, Russia). DC2.4 cells were cultured in RPMI (Gibco, USA) medium with the same supplements. HEK293T/17 cells were cultured in DMEM/F12 (1:1) supplemented with 10% FBS, sodium pyruvate, Glutamax, antibiotic/antimycotic solution, and non-essential amino acids (all reagents from Gibco, USA). To prevent mycoplasma contamination, all cell cultures were constantly tested using the Myco-visor kit (Biolabmix, Novosibirsk, Russia).

### 2.6. Cell Transfection and End-Point Reporter Assays

The day before transfection, HEK293T/17 cells were seeded in 24-well plates at a density of 1.5 × 10^5^ cells/well in DMEM medium (Gibco, USA) supplemented with 10% FBS and antibiotics/antimycotic solution (hereafter, complete medium). The next day, the cells were rinsed with PBS, and 450 µL of DMEM without serum and antibiotics were added to the cells. Lipoplexes were formed in serum-free DMEM by vigorously mixing 0.5 µg of mRNA (in 25 µL of medium) and liposomes at concentrations corresponding to the appropriate N/P ratio (in 25 µL of medium). The resulting mixtures were incubated for 20 min at 25 °C. Lipoplexes were added to the cells and gently stirred. Cells were incubated for 5 h followed by a medium change to complete the DMEM medium. Transgene expression was assessed at times indicated by flow cytometry (for mRNA encoding mKate2) or luminescence measurement (for mRNA encoding Fluc2).

For the flow cytometry assay, cells were detached with TrypLE (Gibco, USA), centrifuged for 5 min at 500× *g*, washed with PBS, and resuspended in 1mL of PBS containing 0.5% FBS. To assess mKate2 expression levels, 10,000 events per sample were processed using BD FACSCanto II flow cytometer (BD Bioscienses, Franklin Lakes, NJ, USA). Transfection efficiency was measured using flow cytometry based on two parameters: transfection percent (as the percentage of mKate2-positive cells) and mean fluorescence intensity (MFI, as the mean value of fluorescence intensity for the gated cell population). The results were analyzed using FlowJo v.10.0.7 software and presented as the mean ± SD obtained from three replicates.

For end-point analysis of Fluc2 expression, 24 h after transfection, the medium was removed, and 200 μL of ice-cold Luciferase Assay Buffer (25 mM Tris-HCl pH 7.8; 1% Triton X-100; 5 mM EDTA; 15 mM MgCl_2_; 75 mM NaCl; 2 mM DTT; 2 mM ATP) was added to the cells. Plates were incubated at +4 °C for 20 min. Lysates were transferred to new tubes and centrifuged at 12,000× *g* for 5 min at +4 °C. A total of 190 μL of each supernatant was transferred to the wells of a 24-well plate, and 10 μL of 3 mg/mL D-luciferin potassium salt (Gold Biotechnology, St. Louis, MO, USA) in PBS was added to each well. Luminescence values were measured immediately after D-luciferin addition using ClarioStar Plus (BMG Labtech, Ortenberg, Germany). The obtained data were analyzed using MARS software v.3.45 R5 (BMG Labtech, Germany).

### 2.7. Time Course of EGFP Expression In Vitro

HEK293T cells in 24-well plates, seeded the previous day in the complete DMEM medium with 10% FBS, were transfected at a density of 2.5 × 10^5^ cells per well using 2X3-DOPE or 2X7-DOPE liposomes loaded with 0.2 µg of EGFP-encoding mRNA. The transfection was carried out in accordance with the principles of the “Fleeting mRNA transfection” (FLERT) technique described by Akulich et al. [66], wherein the culture medium remained unchanged, and the cells were kept at 37 °C throughout the entire procedure to prevent the triggering of the stress response. Following transfection, the plate was immediately returned to the incubator. Puromycin (Cayman Chemical, Ann Arbor, MI, USA; 100 mM stock solution in water) was added into the respective wells at specified time points to a final concentration of 1 mM to arrest the reporter mRNA translation completely [67]. Upon completion of the time course, all samples were simultaneously harvested. For this, the medium was replaced by 80 μL of 0.25% Trypsin-EDTA (Paneco) supplemented with 1 mM puromycin, and the plate was incubated for 3 min at 37 °C. Subsequently, 120 μL of DMEM with 10% FBS and 1 mM puromycin was added to each well, followed by thorough pipetting and transfer to a 1.5-mL tube. The percentage of EGFP-positive cells was assessed using a MACSQuant Analyzer flow cytometer (Miltenyi Biotec, North Rhine-Westphalia, Germany). Percentage value was calculated based on a threshold such that the value at 0 h was set at 0.5%. The mean values ± SD were calculated from three replicates.

### 2.8. Time Course of Luciferase Expression In Vitro

One day prior to transfection, HEK293T cells were seeded into white FB/HB 96-well microplates (Greiner, Kremsmünster, Austria) in DMEM supplemented with 10% FBS at 75 μL per well. Sterile water was used to fill the spaces between wells. The next day, when the cells reached approximately 70% confluency, transfection was performed using HBA1-Fluc2 mRNA. The transfected mixtures were prepared by dilution of the appropriate amount of 2X3- or 2X7-containing liposomes (according to the needed N/P ratio) in 12 μL of sterile PBS per one well, followed by the addition of an equal volume of PBS-diluted mRNA (30 ng per well). Additionally, 100 mM D-luciferin (Promega, Madison, WI, USA) was added to the transfection mixtures at a volume of 0.04 μL per well during preparation. Following a 15 min incubation at 25 °C, the transfection mixtures were transferred into the plate wells. Throughout this step, all manipulations were performed according to the FLERT protocol [66], ensuring a rapid and unstressful transfection procedure. Real-time luminescence measurements were carried out for 24–48 h at 37 °C using the CLARIOstar plate reader (BMG Labtech) equipped with an Atmospheric Control Unit to maintain 5% CO_2_, with a signal integration time of 5 s, essentially as described earlier [60,61]. Each transfection was performed at least twice across different cell passages, in triplicate, and the mean values ± SD were calculated.

### 2.9. Time Course of Luciferase Expression In Vivo

For in vivo experiments, 4–6 week-old BALB/c female mice were obtained from the vivarium of the Institute of Chemical Biology and Fundamental Medicine SB RAS (Novosibirsk, Russia). Every study was conducted with a group size of n = 3–4. Animal experiments were performed in accordance with the recommendations for the proper use and care of laboratory mice (ECC Directive 2010/63/EU). All experimental protocols were approved by the Animal Experiments Ethics Committee at the Institute of Cytology and Genetics SB RAS (Novosibirsk, Russia) (Protocol No. 105 on 18.11.2021).

BALB/c mice were intramuscularly (i/m) injected with lipoplexes in PBS (100 µL per animal) in caudal thigh muscles. Lipoplexes were formed spontaneously in PBS after 10 µg of Fluc2 mRNA was mixed with a definite type of liposomes at N/P ratio of 6/1 and incubated for 20 min at room temperature. In vivo luciferase expression was assessed 4, 8, 24, 48, 72, 96, 120, 144, and 192 h after lipoplexes administration. Animals were injected intraperitoneally (i/p) with 150 µL (3.6 mg per mouse) of freshly prepared D-luciferin potassium salt (Gold Biotechnology, San Diego, CA, USA) in PBS. After 15 min, the animals were anesthetized with isoflurane (Laboratorios Karizoo, S.A., Barcelona, Spain), and bioluminescence was visualized using IVIS Lumina X5 (PerkinElmer, Waltham, MA, USA) with an exposure time of 3 min. Data were processed using Living Image software v. 4.7.4 (Perkin Elmer, Waltham, MA, USA).

## 3. Results

### 3.1. Physicochemical Characteristics of the 2X3- and 2X7-Containing Liposomes and Lipoplexes

Cationic liposomes were prepared using 2X3 [63] or 2X7 [55] cationic gemini lipids and helper lipid DOPE (1,2-dioleoyl-*sn*-glycero-3-phosphoethanolamine) at different molar ratios of cationic to helper lipid from 1:1 to 1:3 (Figure 1A). The cationic lipids consist of natural biocompatible components such as hydrophobic cholesterol residues and spermine, which served as a polycationic head group. Previously, we have demonstrated that the 2X3 lipid provides efficient delivery of DNA, total RNA, oligodeoxynucleotides, and siRNA in vitro and in vivo [52,53,55,68]. Recently, 2X3 has also exhibited its principal suitability for in vitro mRNA delivery into cultured mammalian cells [54]. The presence of two oxygen atoms in 2X7 structure makes it more soluble in biological fluids. DOPE, a zwitterionic phospholipid with an unsaturated lipid tail (Figure 1A), has been widely recognized as one of the most effective helper lipids for nucleic acid delivery [38,69] that facilitates liposome destabilization under low endolysosomal pH conditions, promotes fusion between the liposome and endosome membrane, and enables efficient cargo release into the cytosol [32,34,70]. When exposed to acidic pH conditions, DOPE induces the formation of a hexagonal bilayer phase, thereby driving a structural transition of the LNP and facilitating the endosomal RNA escape [38,71]. For in vivo experiments, PEG-containing cationic liposomes decorated with 2% mol. of DSPE-PEG_2000_ were used (Figure 1A). The addition of PEG lipid into the liposomes could improve the biodistribution, prolong the circulation time of lipoplexes, and increase the delivery efficiency of liposomes [72,73,74]. DSPE-PEG_2000_ is the most commonly used PEG lipid for shielding LNPs from opsonization. A 2000 Mw PEG unit provided prolonged blood circulation time without significant inhibition of LNP internalization. DSPE is a highly stable membrane anchor because of long and saturated lipid chains [74].

Firstly, we focused on the size and polydispersity index of LNPs, as these factors have been shown to significantly impact their therapeutic efficacy [75]. Complexes of cationic liposomes with mRNA (hereafter, lipoplexes) have been formed at different N/P (nitrogen to phosphate) ratios ranging from 1/1 to 8/1, and at different relative proportions of cationic and helper lipids, as these parameters crucially determine the composition of LNPs and modulate their structure, biological properties, and the efficiency of nucleic acid delivery in both in vitro and in vivo settings (see [32,33,76,77,78,79] and references therein). Additionally, we examined the ζ-potential of the particles, which represents the charge on the nanoparticle surface and plays a crucial role in electrostatic interactions with the cellular membrane. The liposomes formed small particles in the solution with diameters ranging from 80 ± 4 to 110 ± 3 nm and a positive surface charge of about +50 ± 2 mV (Figure 1B). At low N/P ratios, lipoplexes increased in size from 120 ± 1 to 150 ± 1 nm compared to empty liposomes. Their negative surface charge (−10–−20 mV) can be explained by the incomplete neutralization of the negative charges of mRNA phosphate groups by the positive amine groups of cationic lipids. The charge transition point of 2X3-DOPE liposomes, where the ζ-potential of the lipoplexes changed from negative to positive, was observed at N/P ratio of 2/1 (Figure 1B). This result is consistent with data previously published for lipoplexes composed of 2X3-DOPE with plasmid DNA [52,80] or mRNA [54]. Among 2X7-DOPE liposomes, only the composition 2X7-DOPE (1:2) exhibited the same charge transition point. In contrast, 2X7-DOPE (1:1) and 2X7-DOPE (1:3) liposomes had a shifted point to a more positive value of N/P 4/1. Apparently, by altering the concentration of DOPE in the cationic liposomal composition, the fine organization of liposomes can be modified, resulting in a non-linear effect on the charge transition point of the lipoplexes. Further increasing the N/P ratio (excess of cationic liposomes) led to the formation of compact positively charged particles with a diameter ≥ 100 nm and a surface charge of 40–50 mV. However, at the N/P ratio of 8/1, the high PDI values indicate the high heterogeneity of the complexes obtained (PDI 0.4–0.5). For lipid 2X3, an increase in DOPE content led to a decrease in the size of both liposomes themselves and their complexes with mRNA, except the complexes at N/P ratio of 8/1, where the size of which did not depend on the DOPE content. Therefore, an increase in the N/P ratio resulted in the formation of more compact and uniform lipoplexes. 

Microscopic imaging using AFM was performed for the most perspective lipoplexes 2X3(1:3)/mRNA and 2X7(1:3)/mRNA at the N/P ratio of 8/1. As a result, mainly spherical particles with a diameter of about 50 nm were observed. However, larger particles (up to 100 nm) with irregular forms were also detected. Representative AFM images are given in Supplementary Figure S1.

**Figure 2:**
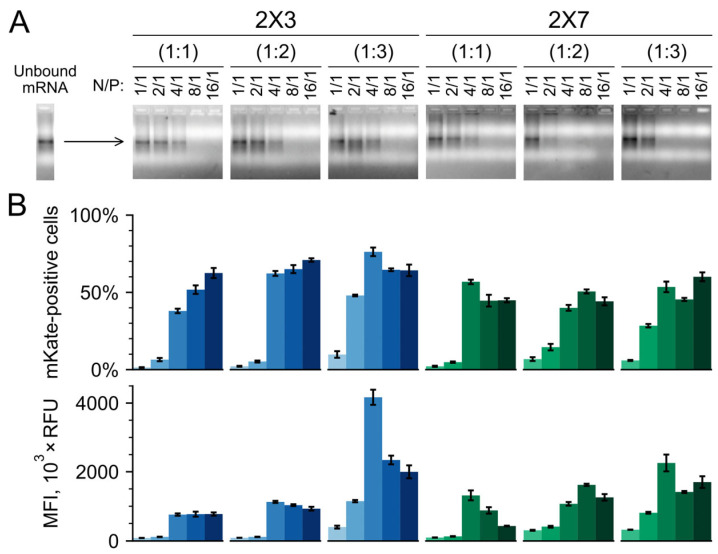
mKate2 mRNA incorporation into 2X3- and 2X7-containing lipoplexes and the efficiency of its delivery into HEK293T/17 cells in a serum-free medium. (**A**) Gel retardation patterns of cationic liposomes complexed with mRNA at various N/P ratios. The complexes were formed for 20 min at 24 °C and then run through a 1.5% agarose gel. The mobility of mRNA (~1200 nt including the poly(A)tail) was visualized by ethidium bromide staining. (**B**) Cell fluorescence at 24 h after 2X3- (blue bars) and 2X7- (green bars) based liposome-mediated delivery of mKate2-encoding mRNA to the HEK293T/17 cells in the serum-free medium (end-point measurement). Lipoplexes were incubated with cells for 5 h in a medium without serum, followed by its replacement by a fresh complete medium. The fluorescence was measured by flow cytometry 24 h post-transfection. Data are presented as mean ± SD. All experiments were run in triplicate for statistical analysis.

The degree of mRNA incorporation into the lipoplexes is an important parameter, as it correlates with mRNA protection from nucleases [81]. To estimate the level of mRNA incorporation into the lipoplexes, a gel retardation assay was performed at different N/P ratios (Figure 2A). mRNA that is fully incorporated into lipoplexes cannot enter the gel due to the large size of the lipoplexes. For all liposomes, it was found that most of the mRNA was in an unbound form at low N/P ratios of 1/1 and 2/1 (large, heterogeneous, slightly positively or negatively charged complexes, Figure 1B). The 2X3-DOPE liposomes fully incorporate mRNA at a relatively high N/P ratio of 8/1, whereas 2X7-DOPE liposomes bound RNA more efficiently and completely incorporated mRNA at an N/P ratio of 4/1 (Figure 2A).

### 3.2. Intracellular Delivery of mRNA with 2X3-DOPE and 2X7-DOPE Liposomes In Vitro

To evaluate the transfection efficiency provided by the 2X3-DOPE and 2X7-DOPE liposomes, we utilized in vitro transcribed mRNA encoding mKate2 fluorescent protein. Following complexation with the liposomes, the mRNA was delivered into cultured human embryonic kidney cells HEK293T/17. In the initial experiment, a short-term 5-h transfection under serum-free conditions was performed. The excess lipoplexes were then removed by medium replacement with fresh complete medium, and the expression of mKate2 was measured 24 h post-transfection (end-point experiment). This strategy allowed for efficient evaluation of mRNA delivery, minimizing potential cytotoxicity from prolonged lipoplex exposure and mitigating negative serum-deficiency impacts on cell viability. It also established a reasonable time window of lipoplex delivery for future in vivo experiments, considering the limited exposure of lipoplexes within body tissues due to systemic circulation, enzymatic degradation, and excretory clearance mechanisms.

We found that mRNA delivery to cultured cells was inefficient at low N/P ratios of 1/1 and 2/1 for both 2X3-DOPE and 2X7-DOPE liposomes. The fluorescence of transfected cells closely resembled the autofluorescence levels of control cells, both in terms of the number of mKate2-positive cells and the level of transgene expression (Figure 2B). This can be attributed to the fact that negatively charged lipoplexes were generated at these N/P ratios (Figure 1B), likely due to the presence of incompletely incorporated RNA molecules on their surface (Figure 2A), which impedes their interaction with the cell membrane. The results demonstrated a gradual enhancement in the efficiency of mRNA delivery with an increase in N/P ratio for all liposomes used. The peak efficiency was achieved at an N/P ratio of 4/1, resulting in 50–70% of cells expressing mKate2 with a mean fluorescence intensity of 1000–4000 RFU. Further elevation of N/P ratios resulted in a slight reduction in the number of fluorescent cells and a more pronounced decrease in their fluorescence intensity. This decline may be due to a reduced mRNA release from endosomes and lipoplexes at high N/P ratios.

The 2X7-DOPE liposomes exhibited mRNA delivery levels comparable to those of the 2X3-DOPE liposomes (Figure 2B). Increasing the amount of DOPE in the liposomal composition mitigated the impact of the N/P ratio on transfection efficiency, with the most effective mRNA delivery observed at a cationic to helper lipid molar ratio of 1:3 for both liposome types (Figure 2B).

It should be noted that cationic liposomes 2X3-DOPE and 2X7-DOPE were nontoxic to cells as even at a concentration of 80 μM, the IC_50_ values were not achieved [52,55].

### 3.3. Kinetics of the Transgene Expression in mRNA-Transfected Cultured Human Cells

**Figure 3:**
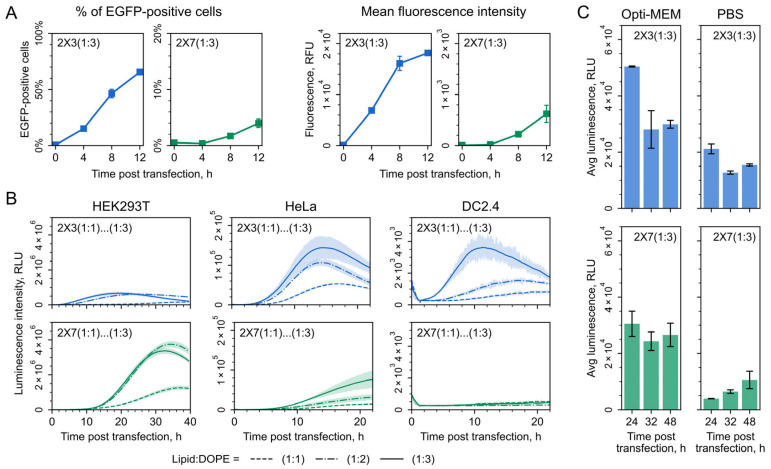
Analysis of mRNA expression dynamics during 2X3- (blue lines) and 2X7- (green lines) liposome-mediated delivery to cultured cells in vitro. (**A**) Time course of the proportion of HEK293T cells transfected with EGFP-encoding mRNA, evaluated by flow cytometry. (**B**) Continuously measured expression of an mRNA coding for firefly luciferase (Fluc) in different cell lines: HEK293T, HeLa, and DC2.4. For transfection, 2X3- and 2X7-containing liposomes with N/P ratio of 10 and indicated cationic lipid to DOPE ratios were used. Luciferase activity curves represent the mean (lines) and SD (shaded regions) of three technical replicates. (**C**) The activity of luciferase in HEK293T/17 cells transfected with lipoplexes 2X3-DOPE (1:3)/Fluc (blue bars) or 2X7-DOPE (1:3)/Fluc (green bars) formed in Opti-MEM or PBS. Data are presented as mean ± SD.

While end-point measurements of the reporter activity yielded valuable insights into the efficacy of the lipoplexes in mRNA delivery, a deeper understanding can be gained from analyzing the dynamics of transfection efficiency. Thus, we developed and applied a flow cytometry-based assay to monitor the evolving proportion of transfected cells over time. At the beginning of the experiment, cells cultured in the same multiwell plate were simultaneously transfected with EGFP-encoding capped and polyadenylated mRNA combined with either 2X3-DOPE or 2X7-DOPE liposomes. Importantly, the transfection procedures were carried out in the complete growth medium and in accordance with the principles of the “Fleeting mRNA transfection” (FLERT) approach to minimize cellular stress. Then, the addition of puromycin was employed at specified intervals to halt the accumulation of EGFP protein (see Materials and Methods for details). Upon the completion of the time course, all samples were concurrently collected for flow cytometry analysis. The results revealed a continuous increase in the percentage of EGFP-positive cells up to 24 to 48 h post-transfection, with an intriguing delay in the appearance of transfected cells in the case of the 2X7-containing lipoplexes (Figure 3A).

We then took another approach, implementing real-time monitoring of luciferase activity in living cells [60,61]. Combined with FLERT principles, this assay enabled a detailed analysis of mRNA expression dynamics at minute resolution, revealing additional insights into intracellular mRNA delivery, translation, and decay. HEK293T cells were transfected with the Fluc-encoding mRNA using various N/P (Supplementary Figure S2) and cationic to helper lipid ratios (Figure 3B). Luciferase activity was continuously measured over 48 h in a temperature-controlled plate reader with CO_2_ supply, as described previously [60,61], with lipoplexes constantly present in the medium throughout the experiment, ensuring continuous mRNA delivery to the cells.

The results of continuous luminescence measurements revealed efficient and prolonged mRNA expression facilitated by the tested lipoplexes. The luciferase activity peaked at ~20–25 h (2X3) or ~30–35 h (2X7) post-transfection, followed by a gradual decline in luminescence, presumably due to mRNA degradation and enzyme turnover. Intriguingly, both 2X3- and 2X7-containing lipoplexes exhibited an unusual delay in the emergence of the luciferase signal, which was particularly pronounced in the case of 2X7 (Figure 3B). This delay had not been observed previously with other delivery systems [59,60,61]. We repeated the experiment using cell lines of different origins, specifically the human cervical carcinoma (HeLa) and mouse dendritic (DC2.4) cells. In both cases, the delay was consistently observed (Figure 3B). 

Importantly, in all cell cultures tested, delivery efficiency increased with an increasing proportion of the helper lipid, with the optimal cationic lipid to DOPE ratio being 1:3, consistently with the results of the earlier end-point delivery experiment (Figure 2B). In certain instances, this ratio also had a slight effect on the dynamics of the expression (e.g., compare 2X3-DOPE samples at 1:1 and 1:3 in HeLa cells in Figure 3B).

Finally, we investigated whether the difference in the kinetics between 2X3-DOPE and 2X7-DOPE lipoplexes observed during continuous transfection can affect the results of transfection when cells are exposed to lipoplexes for only 5 h followed by medium replacement (as described in the previous section). Thus, we performed transfection under these conditions and measured luciferase activity at 24, 32, and 48 h after the addition of the lipoplexes (Figure 3C). As the properties of lipoplexes can be significantly affected by the medium in which they are formed, in this experiment, we also assessed whether lipoplexes formed in a serum-free medium (used in all previous in vitro experiments) are more effective than those formed in a PBS buffer (which is more suitable for subsequent in vivo studies).

Notably, lipoplexes prepared in serum-free medium exhibited more effective mRNA delivery compared to those formed in PBS buffer, as they displayed enhanced luciferase signals at all times tested (Figure 3C). For 2X3-DOPE lipoplexes, peak reporter expression was observed at 24 h post-transfection across both medium types, with a subsequent decline by 20–30% and maintaining a stable level over the following day. Conversely, 2X7-DOPE lipoplexes in medium sustained luciferase expression for 48 h without significant variation between time points (Figure 3C). However, when prepared in PBS, they exhibited a progressive accumulation of the luciferase signal over 48 h, mirroring the kinetic pattern observed during continuous long-term transfection (Figure 3C). This extended trajectory of progressive signal accumulation may be associated with a more gradual mRNA release from lipoplexes/endosomes in the case of 2X7-DOPE compared to 2X3-DOPE.

Thus, the results of in vitro experiments likely support the efficient cellular uptake and rapid endosomal release of mRNA facilitated by 2X3-DOPE/mRNA lipoplexes, typically yielding maximum reporter expression around 24 h post-transfection. While 2X7-DOPE liposomes exhibited lower efficiency, they sustained prolonged reporter production with slower kinetics, potentially implying delayed endosomal escape and/or lipoplex disassembly. The higher mRNA release capability of 2X3-DOPE lipoplexes indirectly arises from the results of the gel retardation assay (Figure 2A), where 2X3-DOPE at an N/P ratio of 4/1 could not allow the full amount of RNA binding, while 2X7-DOPE liposomes fully complexed the nucleic acid under the same N/P conditions. However, these observations require further investigation.

### 3.4. Delivery of mRNA with 2X3-DOPE and 2X7-DOPE Liposomes In Vivo

To assess the efficacy of liposomes in delivering mRNA to mouse tissues in vivo, we included 2% mol. of the commonly used PEGylated lipid DSPE-PEG_2000_ in the liposomal composition (Figure 1A). PEGylation of liposomes is essential for extending the circulation time of the lipoplexes in the bloodstream and preventing rapid clearance, thereby leading to enhanced mRNA expression in vivo [82]. Liposomes composed of cationic lipids 2X3 or 2X7 and DOPE at 1:3 molar ratio were chosen for in vivo experiments, as they have shown maximum efficacy among all tested liposomes in facilitating mRNA transfer into mammalian cells in vitro (Figure 2 and Figure 3).

The sizes of empty PEGylated liposomes as well as their lipoplexes with mKate2 mRNA formed in water at various N/P ratios were assessed using dynamic light scattering (Figure 1B). The results indicated that PEGylation of liposomes did not significantly increase their sizes compared to the original non-PEGylated liposomes. The lipoplexes 2X3-DOPE-DSPE-PEG_2000_/mKate2 exhibited a size range of approximately 100–140 nm and had a PDI value of ~0.25 for all tested N/P ratios. The lipoplexes 2X7-DOPE-DSPE-PEG_2000_/mKate2 had larger sizes ranging from 200 to 600 nm, as well as higher PDI values ranging from 0.40 to 0.55. Both types of lipoplexes exhibit a negative charge at low N/P ratios, consistent with the observations made for non-PEGylated lipoplexes (Figure 1B). The charge transition point was observed at an N/P ratio of 2/1, after which the lipoplexes acquired a positive charge of +30–+ 40 mV. In the case of 2X7-DOPE-DSPE-PEG_2000_ liposomes, large heterogeneous complexes were formed at the charge transition point, with sizes up to 600 nm (Figure 1B), in agreement with previously published data [54,80].

The dependence of liposomes and lipoplex size on the medium was also investigated (Supplementary Figure S3). For PEGylated liposomes 2X3-DOPE, an increase in the liposome size from 100 to 150 nm was observed with higher ionic strength of the solution. In contrast, for 2X7-DOPE liposomes, a size reduction from 180 to 90 nm was observed when ionic strength increased. The sizes of lipoplexes were found to be smaller in deionized water than in higher ionic strength solutions such as PBS. Specifically, the sizes of 2X3-based lipoplexes slightly decreased, while those of 2X7-based lipoplexes increased 2.5-fold in PBS or saline as compared to water (Supplementary Figure S3). These results are consistent with earlier findings [83].

**Figure 4:**
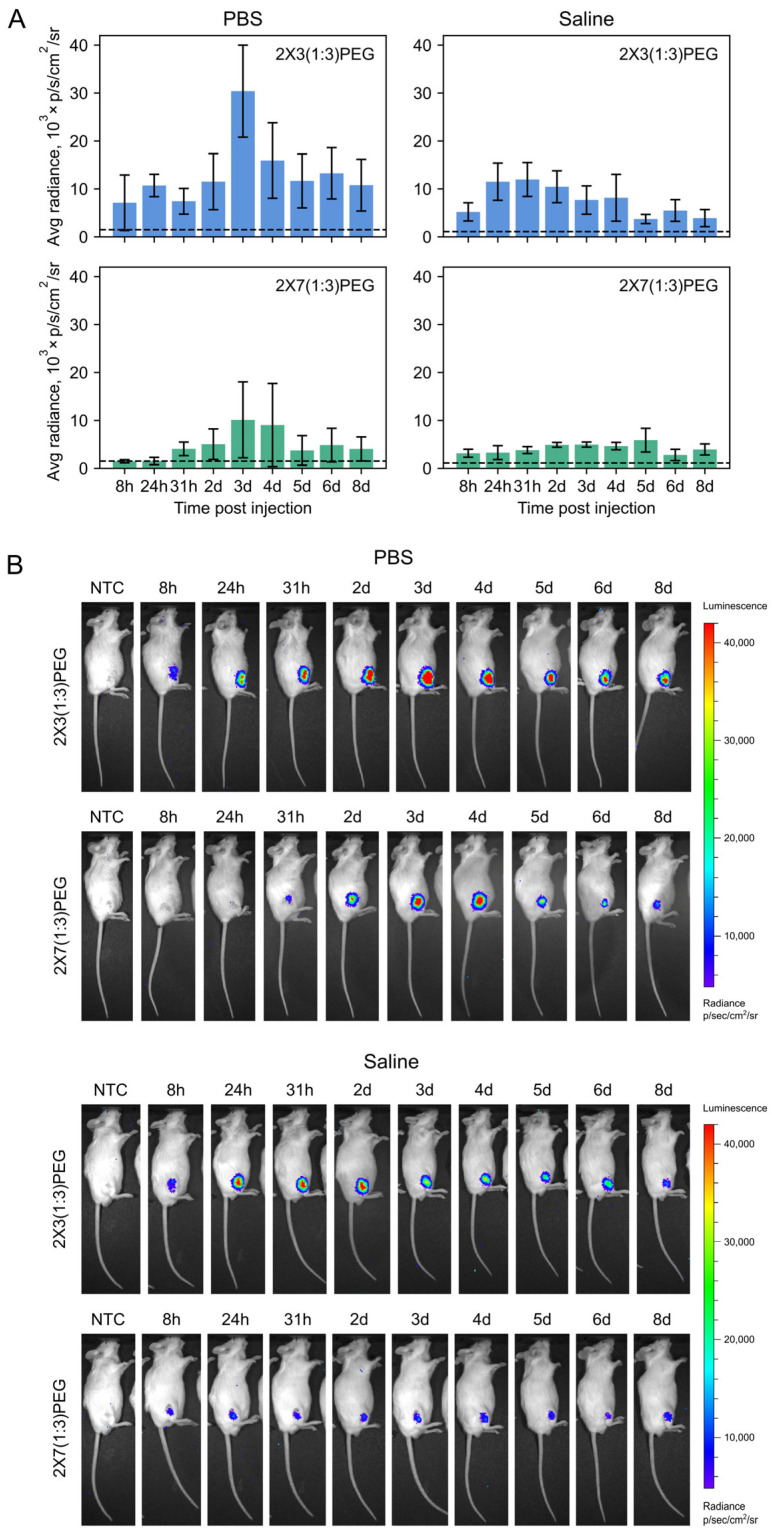
Long-term expression of firefly luciferase in skeletal muscle of mice injected with complexes of 2X3-DOPE-DSPE-PEG_2000_ (blue bars) or 2X7-DOPE-DSPE-PEG_2000_ (green bars) with 10 µg of Fluc mRNA formed at N/P ratio 6/1. The study was conducted with a group size of n = 3–4. Data presented as mean ± SD. (**A**) The average radiance of luciferase luminescence in mice at different time points after the injection of the lipoplexes. (**B**) Representative IVIS images of luciferase luminescence in mice over the course of 8 d after the injection of the lipoplexes. The dashed line in plots represents the background level of luciferase radiance in untreated mice.

To deliver mRNA to mouse tissues in vivo, lipoplexes with mRNA encoding Fluc were formed using 2X3-DOPE-DSPE-PEG_2000_ or 2X7-DOPE- DSPE-PEG_2000_ liposomes at an N/P ratio of 6/1. The lipoplexes were formulated in PBS or saline and administered intramuscularly to mice. mRNA expression was evaluated by measuring luciferase bioluminescence after intraperitoneal injection of D-luciferin at 4, 24, 32, 48 h, and then subsequently every 1 to 2 d until 8 d after the start of the study (Figure 4A).

For both types of liposomes, luciferase activity was detected as early as 4 h after administering mRNA-liposome complexes, irrespective of the medium used for lipoplex formation (Figure 4). Notably, the bioluminescence was observed only at the site of injection, with no detectable signal in the liver or other organs (Figure 4B, Supplementary Figure S4). However, the kinetics of signal accumulation varied depending on the lipoplexes size and the medium used. For both types of liposomes tested, the highest level of luciferase signal level was observed 3 d after initiation of the experiment when PBS was used. The signal then decreased and, by day 8, had reached the same level as on day 2 (Figure 4A). The lower efficiency of 2X7-based lipoplexes in mRNA delivery could potentially be attributed to the larger particle size (Supplementary Figure S3) and a possible slower release of Fluc mRNA from endosomes/lipoplexes as compared to the 2X3-based lipoplexes. When using saline, the 2X3-DOPE-DSPE-PEG_2000_/Fluc lipoplexes showed a peak in luminescence on days 1–2, followed by a gradual decline over the subsequent 4 days. In contrast, 2X7-DOPE-DSPE-PEG_2000_/Fluc lipoplexes formed in saline demonstrated a constant level of reporter expression with a small peak on day 5 (Figure 4A). This result is consistent with the data of transfection on cell lines, where a larger delay between transfection and luminescent signal appearance for 2X7-DOPE liposomes was observed compared to 2X3-DOPE (Figure 3).

Overall, lipoplexes prepared with 2X3-DOPE-DSPE-PEG_2000_ in PBS exhibited higher luminescence intensity. The buffering properties and ionic strength of the medium, as well as the type of cationic lipid, appear to be crucial for the formation of functional lipoplexes. When administered intramuscularly, both PEGylated 2X3-DOPE and 2X7-DOPE liposomal formulations mediated sustained prolonged transgene expression for at least 8 days (Figure 4).

Thus, the results of in vivo experiments demonstrated the successful delivery of mRNA to mouse tissues, resulting in prolonged and efficient transgene expression using 2X3-DOPE and 2X7-DOPE cationic liposomes. The 2X3-DOPE-DSPE-PEG_2000_ liposomes exhibited enhanced delivery efficiency with a relatively faster signal decay, while the 2X7-DOPE-DSPE-PEG_2000_ liposomes displayed a more gradual and prolonged accumulation of the reporter within the tissues.

## 4. Discussion

In this study, we investigated the performance of liposomes containing one of two cationic lipids, 2X3 or 2X7, for the delivery of mRNA to cultured mammalian cells as well as in vivo models. These newly developed delivery systems offer a promising solution to overcome the limitations of commercially available systems by enabling delayed delivery and sustained mRNA expression. To evaluate the performance of our novel LNPs in vitro, we employ real-time monitoring of mRNA expression in living cells of various types. By assessing the kinetics of reporter gene activity, we demonstrate not only the efficient mRNA expression achieved by our LNPs, but also an unprecedented delay (over 10 h, particularly in the case of the 2X7-based system) at the beginning of product accumulation, followed by prolonged reporter protein production. Furthermore, in vivo experiments conducted through intramuscular injections demonstrate the effectiveness of our LNPs in robust luciferase production, with delayed peak activity observed in most cases on day 3 post-injection and prolonged reporter expression up to 9 days. Importantly, we did not observe significant bioluminescence outside of the site of intramuscular injection (i.e., in the liver or other organs). These findings highlight the potential of our mRNA delivery systems for applications requiring sustained protein synthesis, thus opening new avenues for mRNA-based therapies. In particular, the most complex and challenging task in this direction will be the development of individual personalized and “universal” fixed antigen-specific anti-cancer mRNA vaccines.

The diverse clinical applications of mRNA therapy [1,2,3,4,5,11,12,13,14] underscore the vital importance of developing a broad array of effective delivery systems with varied properties. To date, dozens of cationic and ionizable lipid-based systems providing effective mRNA delivery have been described (reviewed in [14,15,16,33,35]). However, although much effort has been made to evaluate their effectiveness, the kinetics of mRNA uptake and expression are much less well studied.

It is widely accepted that RNA-LNPs are internalized by cells shortly after transfection, typically within one hour [24,43,44,45,46,47,48], followed by rapid RNA release from endosomes [23,51]. Our previous data obtained in various cell lines using different delivery systems indicate that reporter activity can be detected as early as 0.25–1.5 h after the addition of lipoplexes to cultured cells [59,60,61]. Although in some instances the observed delay in the appearance of a reporter may extend to ~2 h post-transfection or even longer [40,48,49,50,84], this can typically be attributed to the use of fluorescent or secretory proteins that require maturation or secretory trafficking, as well as potential cellular stress during the transfection process. In our study, we employ luciferase, which becomes active immediately after synthesis [85], and utilize the FLERT procedure to prevent the induction of cell stress [66]. In mice, the expression kinetics after mRNA delivery has also been studied (e.g., see [61,86,87,88,89,90]) and typically found to reach their peak within the first day after injection.

In contrast, with 2X3- and 2X7-based lipoplexes, the start of reporter expression in cultures of cells was detected between 5 to 10 h after transfection. Further incubation of the cells with 2X3-DOPE/Fluc lipoplexes resulted in a significant increase in luciferase expression over the next 10–20 h, depending on lipid composition and cell line. Notably, 2X7-DOPE(1:3) liposomes exhibited especially marked delay and prolonged luciferase expression, commencing at 10–12 h and peaking at 30–40 h post-transfection. Remarkably, this delayed transgene production profile was mirrored in living mice, with luciferase activity peaking at 3 days post-injection (Figure 4B).

Our findings raise questions about the mechanisms underlying mRNA-LNP uptake and subsequent mRNA release [15,17,18,19,20,25,30], which are likely to be specific for 2X3- and 2X7-based lipoplexes. This may be the subject of future investigation. In addition, the unique delayed and prolonged mRNA expression pattern imparted by these lipids could potentially have valuable implications for specific clinical applications.

Despite the significant emergency leap in the development of mRNA technologies and the breakthrough transition from fundamental research and development to the direct implementation of mRNA drugs, a number of questions remain that should be studied, and work on creating new options for delivery vehicles and studying their properties is aimed at solving such questions. Unresolved issues include side effects associated with the unwanted spread of LNPs to other organs and a sufficiently high dose of mRNA drugs used. New delivery systems that have lower toxicity, allow localization of the expression of the gene of interest, and ensure smooth long-term expression may turn out to be a more tunable agent for delivering mRNA.

## 5. Conclusions

In conclusion, our study demonstrated the successful delivery of mRNA to cultured mammalian cells and living mouse tissues using liposomes containing the cationic lipids 2X3 and 2X7. Through real-time monitoring in vitro and long-term bioluminescence tracking in vivo, we comprehensively evaluated the kinetics of reporter gene activity and found that both types of LNPs exhibited a remarkable delay and prolonged mRNA expression for up to several dozen hours in both in vitro and in vivo settings. Notably, in mice, the reporter expression was limited to the site of intramuscular injection. These findings present a promising avenue for prophylactic clinical applications that require sustained synthesis and a continuous supply of therapeutic or defensive proteins.

## Data Availability

Data will be made available on request.

## Supplementary Materials

The following supporting information can be downloaded at: https://www.mdpi.com/article/10.3390/pharmaceutics16050684/s1, Figure S1: Representative AFM images of lipoplexes 2X3(1:3)/mRNA and 2X7(1:3)/mRNA at the N/P ratio 10 of 8/1; Figure S2: Transfection of HEK293T with the Fluc-encoding mRNA using various N/P; Figure S3: The dependence of hydrodynamic diameters of liposomes and their lipoplexes with mKate2 22 mRNA on the medium; Figure S4: Expression of firefly luciferase in skeletal muscle of mice injected i/m and s/c with complexes of 2X3-DOPE-DSPE-PEG2000 with 10 μg Fluc mRNA formed at N/P ratio 6/1 in PBS or Saline with no signal from liver and other organs.
