# Supplementary material for: Novel Efficient Lipid-Based Delivery Systems Enable a Delayed Uptake and Sustained Expression of mRNA in Human Cells and Mouse Tissues
Source: Pharmaceutics. 2024 May 19;16(5):684. doi: 10.3390/pharmaceutics16050684 (PMC11125954; doi:10.3390/pharmaceutics16050684)
Supplement: Supplementary file 1 [file pharmaceutics-16-00684-s001.zip › pharmaceutics-2995510-supplementary.pdf]

pharmaceutics

MDPI

Novel Efficient Lipid-Based Delivery Systems Enable a Delayed Uptake and Sustained Expression of mRNA in Human Cells and Mouse Tissues

Artem G. Fedorovskiy, Denis N. Antropov, Anton S. Dome, Pavel A. Puchkov, Daria M. Makarova, Maria V. Konopleva, Anastasiya M. Matveeva, Eugenia A. Panova, Elena V. Shmendel, Mikhail A. Maslov, Sergey E. Dmitriev, Grigory A. Stepanov, and Oleg V. Markov

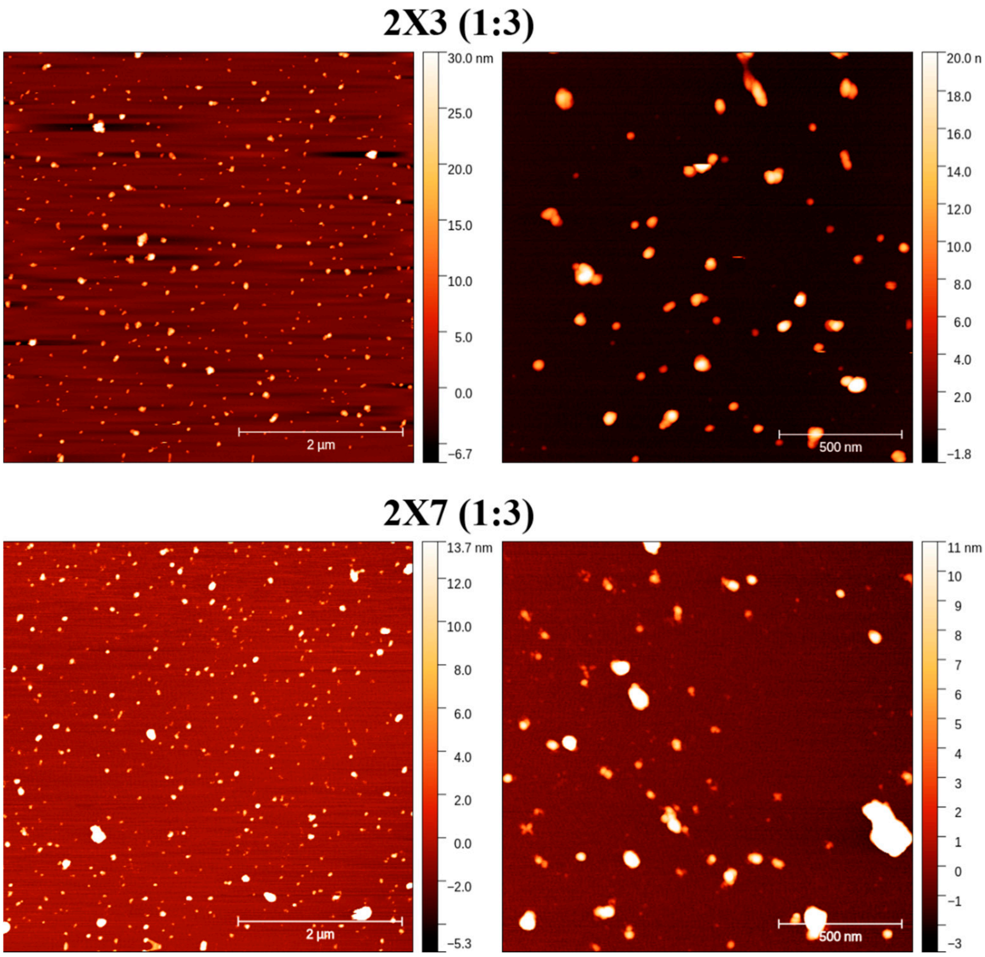

Supplementary Figure S1. Representative AFM images of lipoplexes 2X3(1:3)/mRNA and 2X7(1:3)/mRNA at the N/P ratio of 8/1. The scale bars are 2 µm (left) and 500 µm (right). Pseudo-color rulers reflect the particle height distribution in each sample.

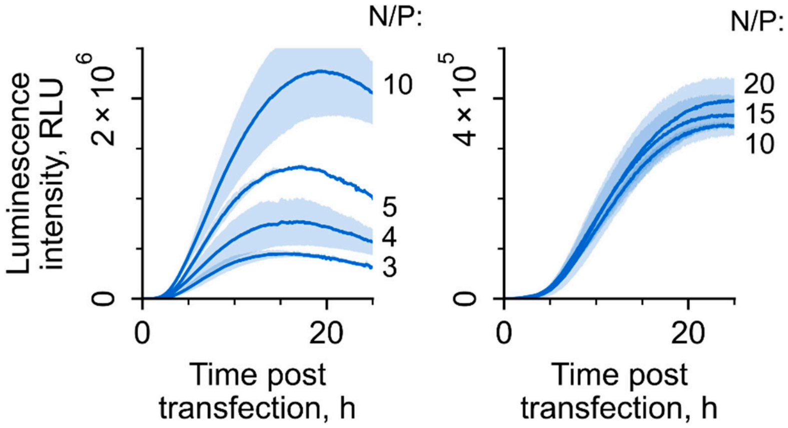

Supplementary Figure S2. Transfection of HEK293T with the Fluc-encoding mRNA using various N/P.

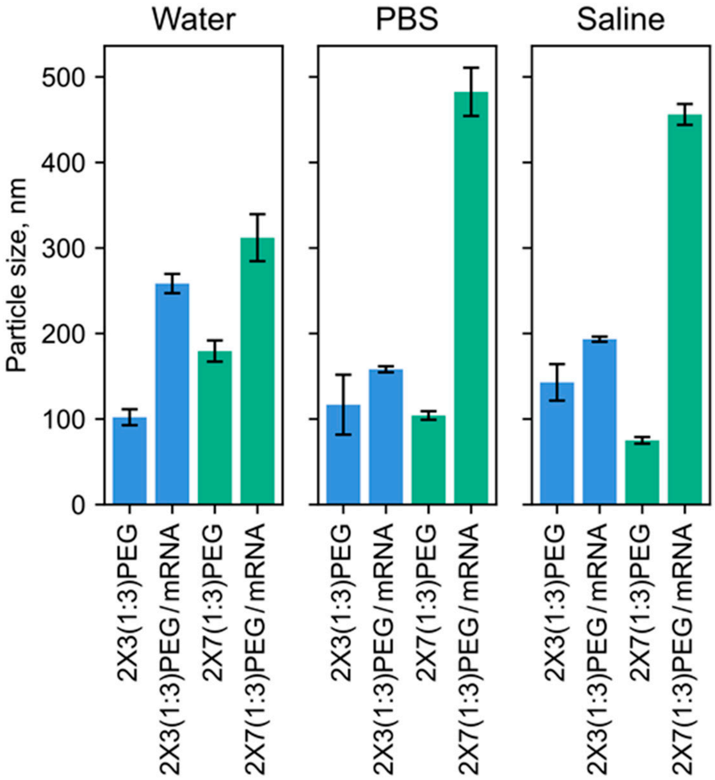

Supplementary Figure S3. The dependence of hydrodynamic diameters of liposomes and their lipoplexes with mKate2 mRNA on the medium. Data are presented as mean±SD.

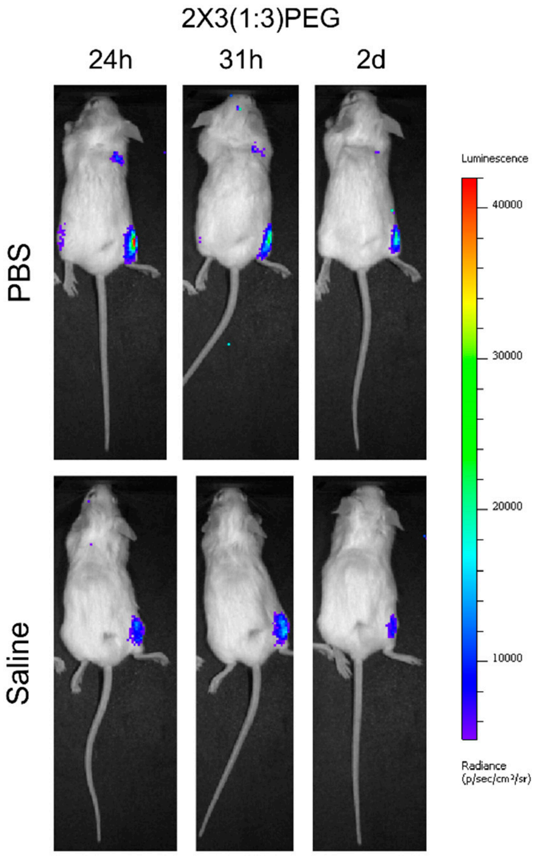

Supplementary Figure S4. Expression of firefly luciferase in skeletal muscle of mice injected i/m and s/c with complexes of 2X3-DOPE-DSPE-PEG$_{2000}$ with 10 μg Fluc mRNA formed at N/P ratio 6/1 in PBS or Saline with no signal from liver and other organs.
